# Natural polyphenol self-assembled pH-responsive nanoparticles loaded into reversible hydrogel to inhibit oral bacterial activity

**DOI:** 10.1186/s43556-022-00082-3

**Published:** 2022-09-16

**Authors:** Yunyun Qi, Jinxiang Yang, Yaping Chi, Peng Wen, Zhongying Wang, Shiyi Yu, Rui Xue, Jingmin Fan, Hong Li, Wen Chen, Xinjun Wang, Yan Zhang, Gang Guo, Bo Han

**Affiliations:** 1 School of Pharmacy/School of Food science/Key Laboratory of Xinjiang Phytomedicine Resource and Utilization, Ministry of Education, Shihezi University, Xinjiang, Shihezi 832002 PR China; 2 Department of Pharmacy, Karamay Central Hospital, Karamay, 834000 PR China; 3 State Key Laboratory of Biotherapy and Cancer Center, and Department of Otorhinolaryngology, Head and Neck Surgery, West China Hospital, Sichuan University, and Collaborative Innovation Center for Biotherapy, Chengdu, 610041 PR China; 4 Sinopharm Xinjiang Pharmaceutical Co., Ltd, Urumqi, 830000 PR China

**Keywords:** Natural polyphenols, Oxidative self-polymerization, Nanoparticles, pH sensitive, Anti-bacterial, Anti-oxidation

## Abstract

Periodontitis is one of the most prevalent chronic inflammatory diseases and Polyphenols isolated from Turkish gall play a major role in the treatment of inflammatory diseases for their antibacterial, anti-inflammatory and antioxidant activities. In this work, Turkish Galls effective constituent (TGEC, T) was prepared into nanoparticles (T-NPs) by principle of oxidative self-polymerization. The pH-sensitive T-NPs was encapsulated into thermosensitive type in-situ hydrogel, and 42.29 ± 1.12% of effective constituent from T-NPs were continuously released within 96 h under the periodontitis environment. In addition, the weakly alkaline oral micro-environment of patients with periodontitis is more conducive to the sustained release of effective constituent, which is 10.83% more than that of healthy periodontal environment. The bacteriostatic test showed that T-NPs had stronger antibacterial activity on oral pathogens than that of TGEC. Compared with TGEC, the minimum inhibitory concentration (MIC) of T-NPs against *P. gingivalis* and *A. viscosus* was reduced by 50% and 25%, respectively. Interestingly, T-NPs induced bacteria lysis by promoting the excessive production of ROS without periodontal tissue damage caused by excessive oxidation reaction. In conclusion, a simple method of preparing microspheres with natural polyphenols was developed, which provides beneficial reference for one-step prepared drug carriers from effective components of natural product, likewise the method offers a green and effective solution to synthesis a new adjuvant therapy drugs for treatment of gingivitis associated with periodontal pockets.

**Supplementary Information:**

The online version contains supplementary material available at 10.1186/s43556-022-00082-3.

## Introduction

Periodontitis is one of the most prevalent chronic inflammatory diseases that causes irreversible loss of the tooth-supporting apparatus, including periodontal ligament, cementum, and alveolar bone, affecting over 90% of the world’s population [1]. Currently, the mechanical debridement of bacteria plaque, the antibiotics and anti-inflammatory drugs of treating bacterial infections, and surgery for severe cases are accepted as standard treatment for periodontal disease [2]. However, Scaling and root planing alone may not suffice to completely remove the plaque and predisposing factors in deeper periodontal pockets in cases where surgical therapy cannot be undertaken [3]. Long-term abuse of oral antibiotic will lead to damage to the oral micro-environment [4], bacterial resistance, and systemic side effects [5]. Surgical therapy is effective to periodontitis treatment but often comprises with complicated series of steps and have tissue exposure risk. In this regard, green innovative non-surgical yet efficient alternatives towards periodontal disease are highly desired.

Turkish gall (TG) is one of the tannin-rich insect gall, which has the effects of inhibiting bacteria, relieving pain, diminishing inflammation, reinforcing gums, etc. [6]. Xipayi gingiva made of TG has an excellent protective effect on oral health [7] and has been widely used in Xinjiang Uygur Autonomous Region, China. It has been reported natural extracts gallnut tannins (GTs) as functional components to prepare chitosan/gallnut tannins composite fiber, which can effectively inhibit the growth of *S. aureus* [8]. Moreover, the effective components of TG against oral ulcer and ulcer colitis have been identified, determined, and compounded with Fe^III^ to prepare TGTs-Fe^III^ microcapsules with the therapeutically effects on ulcerative colitis treatment in our previous studies [9], which provided an alternative for the treatment of periodontitis.

With the development of wound dressings for hundreds of years, a vast number of wound dressings have been developed to date [10], such as gauze/bandage [11, 12], hydrogel [13, 14], and foam [15, 16]. Of late, the hydrogel formulations in periodontal applications have gained enormous interest owing to their excellent mucoadhesion properties and ability to maintain controlled drug release profiles [17]. Studies on in situ gel loaded with antibiotics doxycycline or metronidazole have been gradually improved, including preparation process research, performance, antibacterial test, etc. [18, 19]. Besides, the effective treatment of periodontitis requires maintaining drug concentrations above minimum inhibitory concentration (MIC) in the periodontal pocket [20]. Use of polymeric nanoparticles, in this aspect, have generally been explored in drug delivery research for controlled drug release profiles [21].

Various techniques such as oxidative self-polymerization, metal-phenolic network assembly, and layer-by-layer assembly have been developed to prepare functional materials from natural phenolic compounds for treatments, medical supplies, and food packaging field. For example, thin films [22, 23], gels [24] and particles [25, 26] are attractive drug delivery platform because of their higher drug-loading rate, controllable feature, multiple responses, and good biocompatibility. Oxidative self-polymerization assembly can synthesize nanoparticles by the properties of phenolic materials, which has promising prospects in the field of oral care medicine. In particular, different from the common multifunctional nanoscale drug carrier synthesis [27], there is no need for multi-step coupling and purification reactions. Degradable poly-dopamine nanoparticles prepared by oxidative self-polymerization are used as reactive oxygen species (ROS) scavengers to treat a range of diseases caused by ROS, including periodontitis caused by oxidative stress [28]. However, a challenge still remains in researching an efficient application of plant polyphenols in complexity of periodontal environment and enhancing the anti-bacteria effect.

**Fig. 1:**
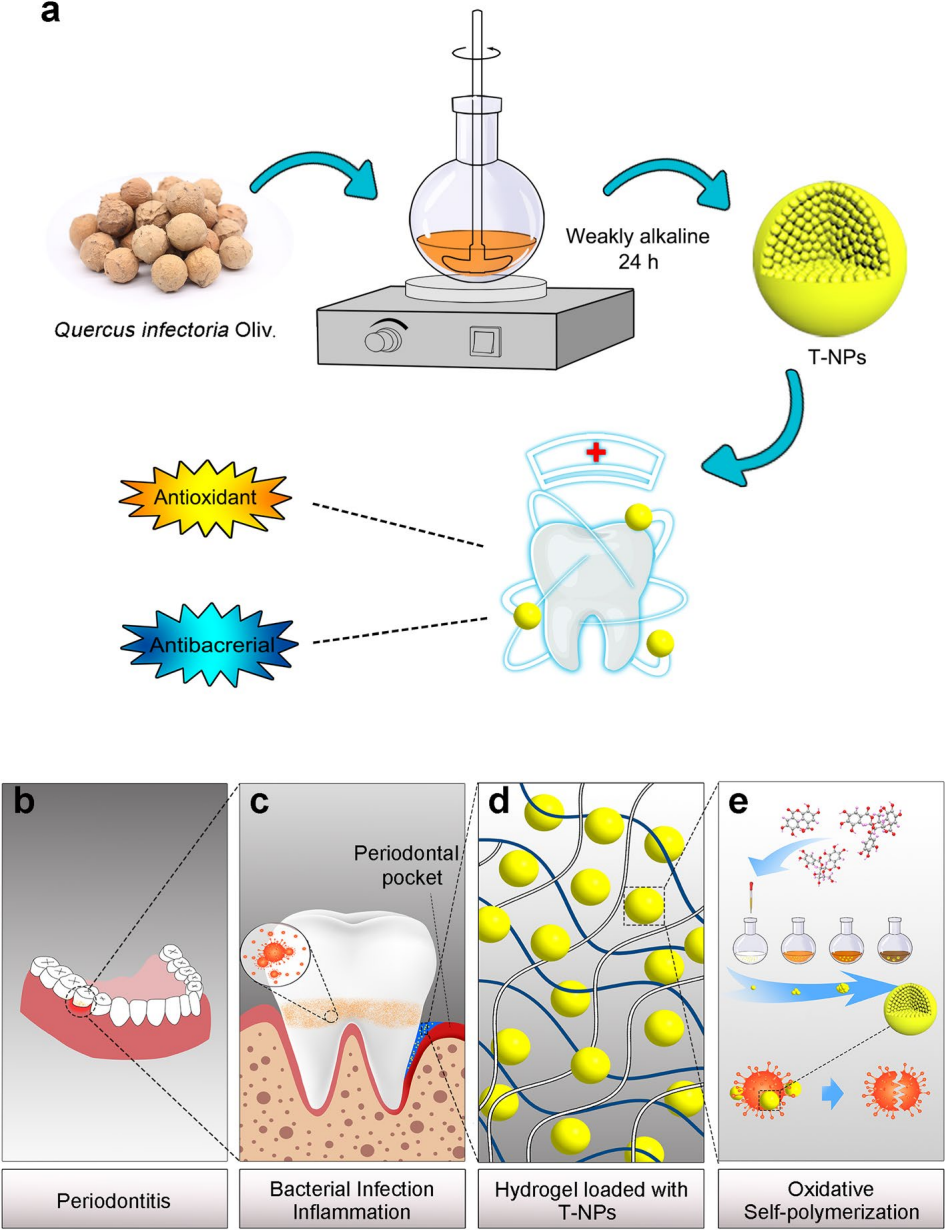
Formation of Turkish Galls effective constituent (TGEC) nanoparticles and preparation of the smart hydrogel loaded with T-NPs for potential application in periodontitis. **a** Formation of TGEC nanoparticles and their antioxidant and antibacterial activity in periodontitis. **b** Schematic representation of periodontitis. **c** Anaerobic bacteria colonize the anaerobic periodontal pocket, which contributed to oral infection and possible results in periodontitis. **d** The smart hydrogel loaded with T-NPs. **e** Preparation of T-NPs by oxidative polymerization, and it exhibited high antibacterial action

In this work, nanoparticles made of natural polyphenol mixture were one-step synthesized by oxidative self-polymerization of effective constituent in TG, and thermosensitive antibiotic platform was rationally designed by the incorporation of T-NPs and poloxamers in situ gel. T-NPs with a beneficial property, which suitable for release in mildly alkaline of oral environment and reach and adhere to the periodontal pocket, and exert its antioxidant and antibacterial effects through sustained release and long-term release (Fig. 1).

## Results

### Chemical composition of TGEC identification

**Fig. 2:**
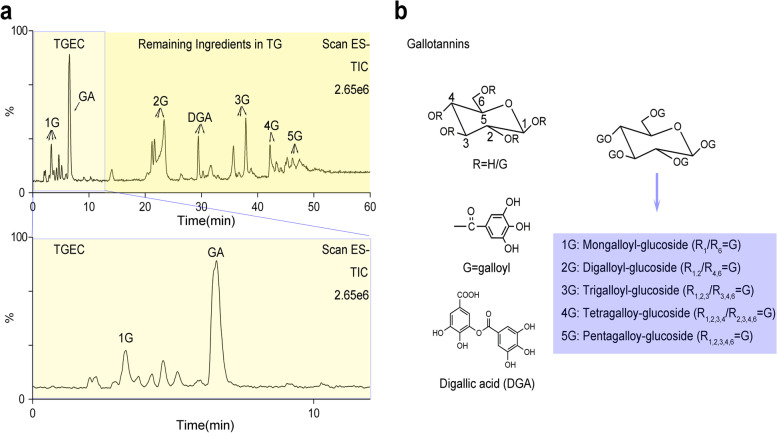
Chemical composition of TGEC identification. **a** LC-MS spectrometry identify of Turkish Galls. **b** Fraction 15% ethanol of Turkish Galls was isolated by polyamide column chromatography and identified by LC-MS

The dry weight ratio of extracts to herbs was 70.12%. The chemical composition in TG and fraction TGEC were identified by LC-MS. The main components were tannins, with the typical total ion chromatogram in negative ion mode (Fig. 2**)**. In the chromatogram of fraction TG, the constituents were identified as gallic acid (*m/z* 169.07), digallic acid (*m/z* 321.09), mono-*O*-galloyl-β-D-glucose (*m/z* 331.20), di-*O*-galloyl-β-D-glucose (*m/z* 483.26), tri-*O*-galloyl-β-D-glucose (*m/z* 635.34), tetra-*O*-galloyl-β-D-glucose (*m/z* 787.24), penta-*O*-galloyl-β-D-glucose (*m/z* 939.27), and hexa-O-galloyl-β-D-glucose (*m/z* 1091.63), respectively. In the chromatogram of fraction TGEC, the constituents were identified as gallic acid (*m/z* 169.07) and mono-*O*-galloyl-β-D-glucose (*m/z* 331.20). Our group had identified the chemical structures of these constituents and discussed the fragmentation regularity [29]. Based on area normalization method, the quantitation result showed that the amount of mono-*O*-galloyl-β-D-glucose and gallic acid was 30.29% area of the total peak area of TGEC components.

### Mechanism discussion of oxidative self-polymerization

Gallic acid nanoparticles (GA-NPs) were identified by LC-MS in negative ion mode, the mass-to-charge radio changed from *m/z* 170 to *m/z* 322 at 2 h of reaction, the synthesis mechanism was inferred that polymer produced by the carboxyl group and the hydroxyl group of different Gallic acid occurring dehydration reaction (Supplementary Fig. 1). At the time of reaction for 4 h, the mass-to-charge radio changed from *m/z* 322 to *m/z* 474, 3 molecules of Gallic acid formed a chain structure by dehydration reaction.

### Characterization of T-NPs

**Fig. 3:**
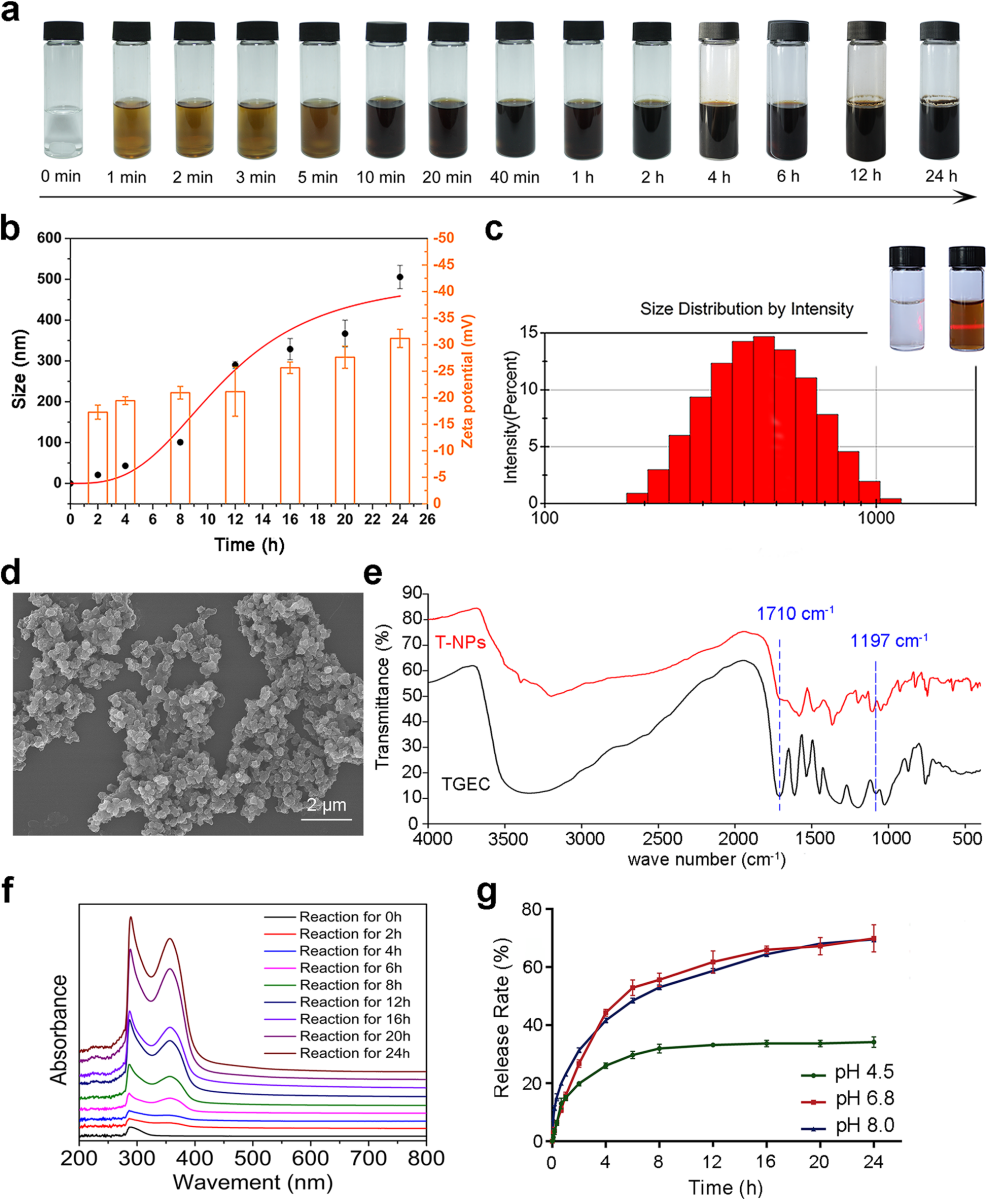
Preparation and characterization of T-NPs. **a** Formation of T-NPs during 24 h. **b** Particle size and zeta potential variation during 24 h. **c** The size distribution of T-NPs at reaction 24 h. The Tyndall effect of TGEC (left) and T-NPs solution (right) at reaction 4 h was shown in inset photo of 3C. d SEM image of T-NPs at reaction 24 h. **e** Infrared spectrum absorption of TGEC (black) and T-NPs (red). **f** The UV-Vis spectrum of TGEC (black curve) and T-NPs (red curve). **g** The accumulated release rate of T-NPs in artificial saliva with different pH values

The forming process of T-NPs was shown in Supplementary Video 1, the solution was clear and colorless before adding the TGEC. The reaction solution gradually changed from pale yellow to brown, and turned dark brown with extension of stirring time when TGEC dispersed into solution (Fig. 3a). A series of Malvern laser granulator measurements on the reaction process were investigated to unveil size distribution quantitatively (Fig. 3b). The size of particle was 20.87 ± 4.92 nm at 2 h of the reaction. At 24 h, the size of particle was increase to 505.63 ± 28.52 nm. Zeta potential of T-NPs from − 17.3 ± 1.33 mV at reaction 2 h to − 31.2 ± 1.72 mV at reaction 24 h. The results were further confirmed by Malvern laser granulator and indicated that the particle size was controlled by reaction time. Size distribution image of T-NPs was shown in Fig. 3c, T-NPs were detected by Tyndall effect when it was added to reaction 4 h. T-NPs were round or nearly round, and its particle size was 571.26 ± 62.81 nm measured by SEM (Fig. 3d). The particles size in consistency with the size measured by Malvin granularity analyzer.

The results of the FT-IR spectroscopy of the TGEC and T-NPs have shown that the characteristic absorption band of TGEC was at 1710 cm^− 1^ and 1197 cm^− 1^ due to the stretching vibration of C=O (ester) and Ar − OH, respectively. After reaction, T-NPs maintained original characteristic bands and had a weaker hydroxyl or associative hydroxyl bond, revealing the possibility of self-polymerization (Fig. 3e). UV-Vis spectrophotometer was used to compare the values products at different reaction time (Fig. 3f). To minimize scattering interference and ensure the absorption spectrum of 800 nm was close to the baseline, sample solutions was diluted and measured by UV. In polymerization process, the new absorbance band occurring at 353 nm, explaining quinone compound had formed in alkaline conditions [30]. Combined with LC-MS spectrometry analysis of GA-NPs, the appearance of benzoquinone structure is a sign of polymer formation in the process of polymerization. Based on the above analysis, T-NPs with circular or nearly circular structure were formed by oxidative self-polymerization of TGEC, which using a greener synthetic route.

### Characterization of hydrogel loaded with T-NPs

**Fig. 4:**
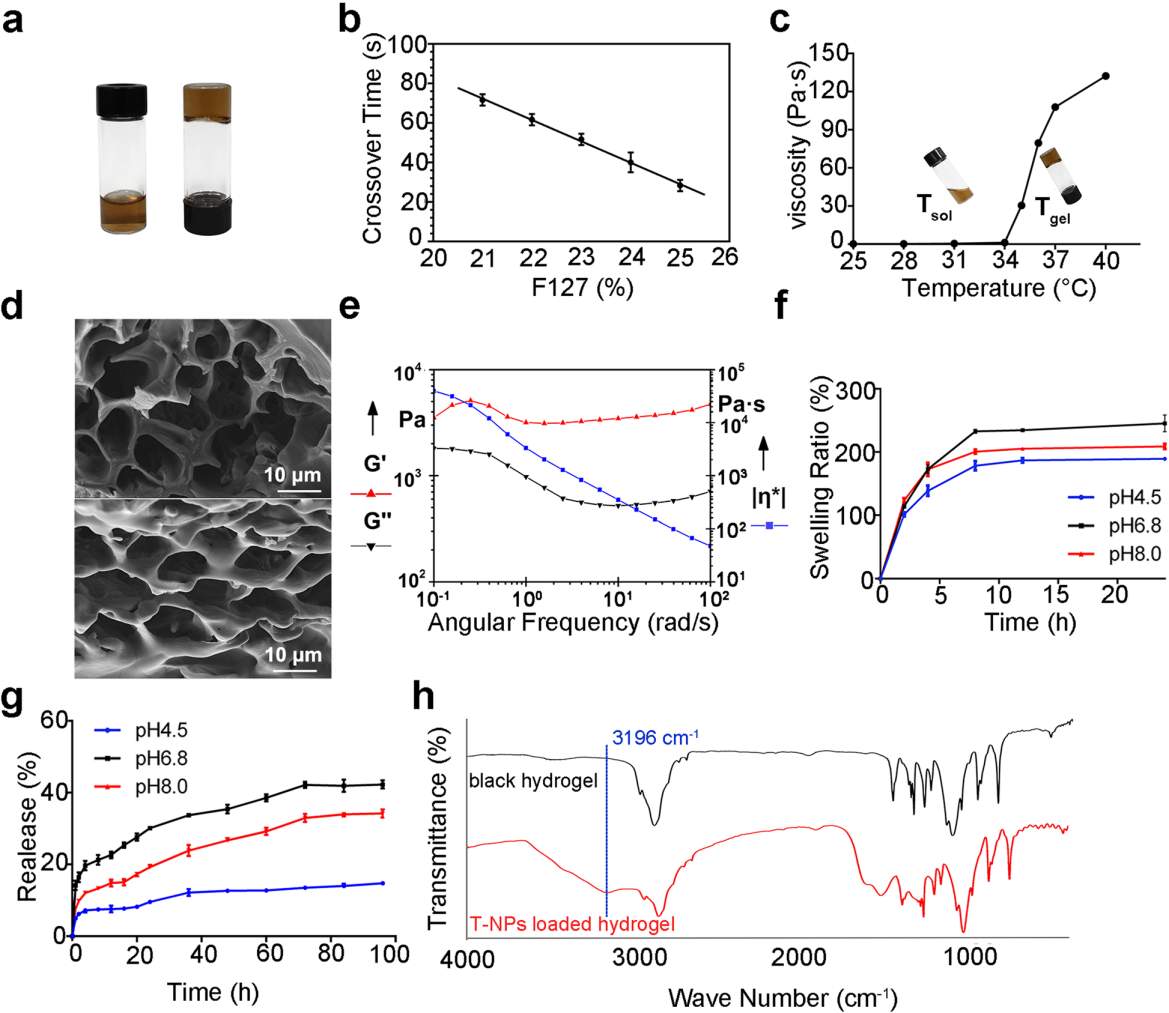
Characterization and property of hydrogel loaded with T-NPs. **a** Photograph of T-NPs loaded hydrogel in sol and gel state respectively at room temperature (left) and at 37.0 °C (Right). **b** The influence of F127 content on gelation time. **c** Viscosity of T-NPs-loaded hydrogel in different temperature. **d** SEM image of blank hydrogel (up) and T-NPs-loaded hydrogel (down). **e** Rheology analysis of T-NPs loaded hydrogel. **f** Swelling test of T-NPs loaded hydrogel in artificial saliva with different pH values. **g** The accumulated release rate of T-NPs loaded hydrogel in artificial saliva with different pH values on 96 h. **h** Infrared spectrum absorption of blank hydrogel (black) and T-NPs loaded hydrogel (red)

Due to the limited ability of thermosensitive gels to control drug release, the potential of controlling the drug release via the size of the T-NPs provided a new strategy for in situ release kinetics of the periodontal delivery system. To investigate the characterization and properties of hydrogel loaded with T-NPs, a series of experiments were conducted. A state changed from sol phase to gel phase as the temperature increased from 25 °C to 37.0 ± 0.5 °C for T-NPs loaded hydrogel (Fig. 4a). The gelation time of T-NPs-loaded hydrogel from sol state to gel state at 37.0 ± 0.5 °C is gradually shortened with the increase the content of F127 (Fig. 4b). The formula containing 25% F127 and 1% F68 as hydrogel matrix, and its optimum formulation for rapid coagulation at 37.0 ± 0.5 °C was explored. The viscosity of T-NPs loaded hydrogel was reached to 107.8 Pa·s at 37.0 ± 0.5 °C, which was significantly higher than the initial solution state of 0.180 Pa·s (Fig. 4c). In addition, Microporous morphology was observed for blank gel and T-NPs loaded gel by SEM, the pores became slightly larger after loading with T-NPs and round bulges was observed in the pores, indicating that T-NPs have been loaded on the gel (Fig. 4d). The rheological property of the gel was measured at 37.0 ± 0.5 °C. Under the conditions of strain force γ = 0.1% and the frequency range of 0.1 ~ 100 Hz, G′ values are greater than G′′ (Fig. 4e), indicating that hydrogels were more viscous than elastic and remain solidified in this range. T-NPs loaded hydrogel had good swelling properties at artificial saliva with different pH values. After swelling in artificial saliva with pH 6.8 for 24 h, the maximum swelling ratio reached 234.62 ± 0.34% (Fig. 4f). Compared with the blank gel, the characteristic absorption band of T-NPs was found on the FT-IR spectroscopy of T-NPs-loaded hydrogel in 3196 cm^− 1^, the red shift of OH^−^ stretching vibration frequencies reveals that the T-NPs were added to the hydrogel and may take place physical mixture (Fig. 4h). The hydrogels investigated possessed fine viscoelasticity, swelling property and can quickly gelation in less than 30 ± 2 s at body temperature. These properties gave the hydrogel loaded with T-NPs favorable potential application prospect in a periodontal inflammatory environment.

### Release capability of T-NPs from hydrogel

T-NPs and T-NPs loaded hydrogel release were assessed under different pH artificial saliva. The periodontal pocket is a pathological deepening of the sulcus. Formation of an anaerobic environment, reduction of local redox potential, and production of alkaline reducing substances such as histamine by oral anaerobic microorganisms also make the pH value of periodontal pockets into weakly alkaline [31]. At the time of reaction for 24 h, the release efficiency of T-NPs in artificial saliva with pH values at 4.5, 6.8 and 8.0 were 34.16 ± 1.20%, 69.89 ± 3.21% and 69.53 ± 1.77%, respectively (Fig. 3g); the release efficiencies of T-NPs from hydrogel in artificial saliva with pH values of 4.5, 6.8 and 8.0 were 9.59 ± 0.43%, 19.29 ± 0.57% and 30.12 ± 0.57%. When released continuously for 4 days, the release efficiency was estimated to be about 14.78 ± 0.22%, 34.20 ± 1.14% and 42.29 ± 1.12%, respectively (Fig. 4g). The potential of the T-NPs were measured under different pH conditions of artificial saliva (Supplementary Fig. 2) and found that the maximum absolute value of potential was 41.6 ± 1.1 mV at pH 6.8, 39.5 ± 0.7 mV at pH 8.0 and 38.9 ± 1.4 mV at pH 4.5. Compared with acidic environment, T-NPs were more stable in neutral or weakly alkaline environment, which was conducive to the sustained release of drugs.

### Antioxidant capacity analysis of T-NPs

**Fig. 5:**
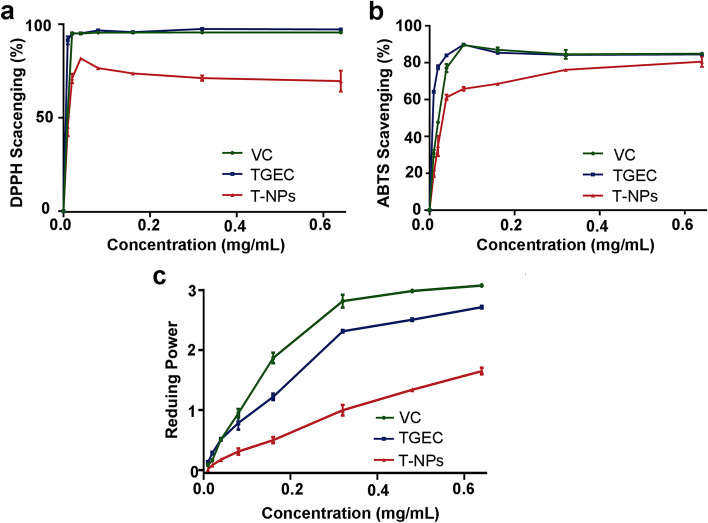
Anti-oxidation capacity of T-NPs. **a** DPPH radical scavenging activity of T-NPs. **b** ABTS radical scavenging activity of T-NPs. **c** Reducing power of T-NPs

Antioxidants activities of T-NPs were evaluated by using DPPH radical scavenging assay, ABTS radical scavenging assay and reduce power. As shown in Fig. 5a-c. TGEC exhibited good radical scavenging activities at a concentration range of 0 ~ 0.64 mg/mL. The DPPH radical scavenging rate of T-NPs was closed to 72.75%. Compared with TGEC, the DPPH radical scavenging rate of T-NPs slightly decreased, because the specific surface area and contact area of T-NPs are smaller, and their sustained release ability may be longer than that of TGEC. Hydroxyl groups are used in the process of oxidative self-polymerization, but still retain most of the antioxidant capacity of TGEC. The oxidative polymerization of TGEC had smaller influence on ABTS radical scavenging ability, and T-NPs still reserved good activity in ABTS radical scavenge. In contrast, the reduce power of TGEC, T-NPs and VC were 2.72, 1.65 and 3.08, respectively. Although the reduction power of T-NPs had decreased, it still had good reduction ability. The results of antioxidant test showed that T-NPs had dose-dependent in the concentration range of 0 ~ 0.64 mg/mL. T-NPs still retained over 50% the antioxidant ability of TGEC and might play an important role in the progression of ROS-related diseases.

### Evaluation of T-NPs antibacterial test

**Fig. 6:**
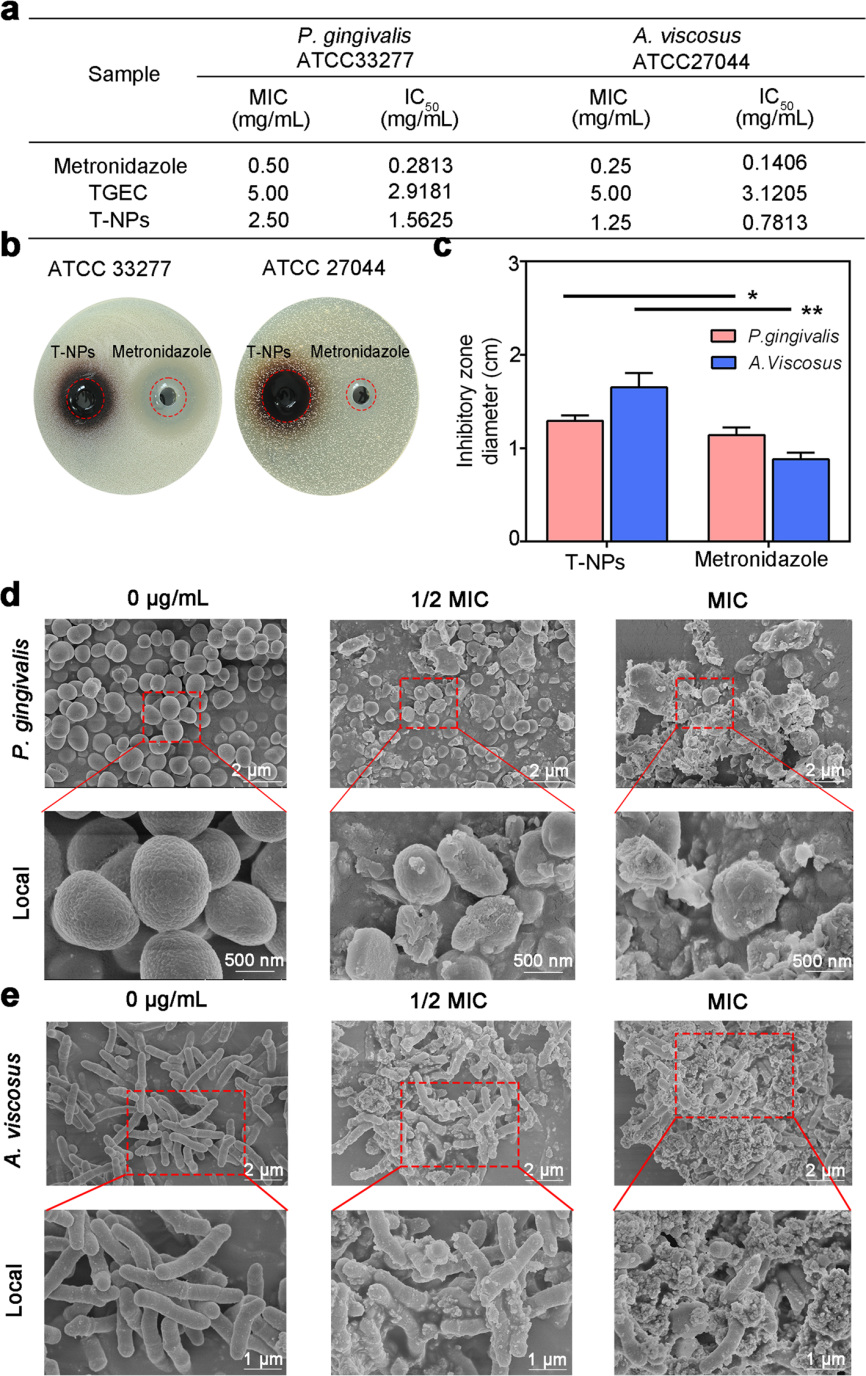
Antibacterial property of T-NPs. **a** MIC and IC_50_ of samples on *P. gingivalis* or *A. viscosus*. **b** Inhibition zone image of T-NPs on *P. gingivalis* or *A. viscosus*. **c** Quantification of inhibition zone. **d** *P. gingivalis* and **e** *A. viscosus* cultured in the medium with 1/2 MIC of T-NPs, MIC of T-NPs or without T-NPs. **d** SEM images of *P. gingivalis* and **e** *A. viscosus* cultured in the medium with 1/2 MIC of T-NPs, MIC of T-NPs or without T-NPs

Periodontitis is one of the most prevalent oral diseases in humans. The formation of plaque biofilm is the main pathogenic factor of periodontal disease, which *Actinomyces viscosus* (*A. viscosus*) can mediate the aggregation of various oral bacteria in dental plaque [32]. Moreover, *Porphyromonas gingivalis* (*P. gingivalis*) is a major etiologic agent in the initiation and progression of chronic periodontitis among the various organisms that reside in theoral tissue [33]. These two bacteria were selected as experimental subjects to investigate the antibacterial effect of nanoparticles. The MIC values of metronidazole against *P. gingivalis* and *A. viscosus* were the lowest, which were 0.50 and 0.25 mg/mL, respectively. The MIC values of TGEC against *P. gingivalis* and *A.viscosus* were both 5.00 mg/mL. The MIC values of T-NPs against *P. gingivalis* and *A.viscosus* were 2.50 and 1.25 mg/mL, respectively (Fig. 6a). The results indicated the original antibacterial activity of TGEC was improved through oxidative self-polymerization. The inhibitory zone of T-NPs on *P. gingivalis* and *A.viscosus* were 12.91 ± 0.06 mm and 16.50 ± 0.15 mm, respectively, while the inhibitory zone of metronidazole (5 mg/mL) on *P. gingivalis* and *A. viscosus* were 11.4 ± 0.08 mm and 8.8 ± 0.07 mm (Fig. 6b-c). The result showed that the antibacterial activity of T-NPs was stronger than metronidazole at its commonly used dose.

The changes of the morphology and membrane integrity of bacterial treated with MIC and 1/2 MIC T-NPs or without T-NPs were further determined by FE-SEM in order to understand the antibacterial mechanism of T-NPs. Bacteria cells of both *P. gingivalis* and *A. viscosus* cultured without T-NPs were observed with no cell death and membrane damages. By contrast, the bacteria were partly deformed by treated with T-NPs at a concentration of 1/2 MIC, most bacterial suffered showed a prevalent membrane damage and cytoplasm leakage. Both bacteria cells suffered from prevalent cell lysis, indicating a severe membrane disruption and cytoplasm leakage when cultured in the presence of MIC T-NPs (Fig. 6d-e). These results demonstrated that the destroyed degree of bacterial morphology was connected to the concentration of T-NPs, and suggested that cell inhibitory mechanism might due to membrane disruption.

**Fig. 7:**
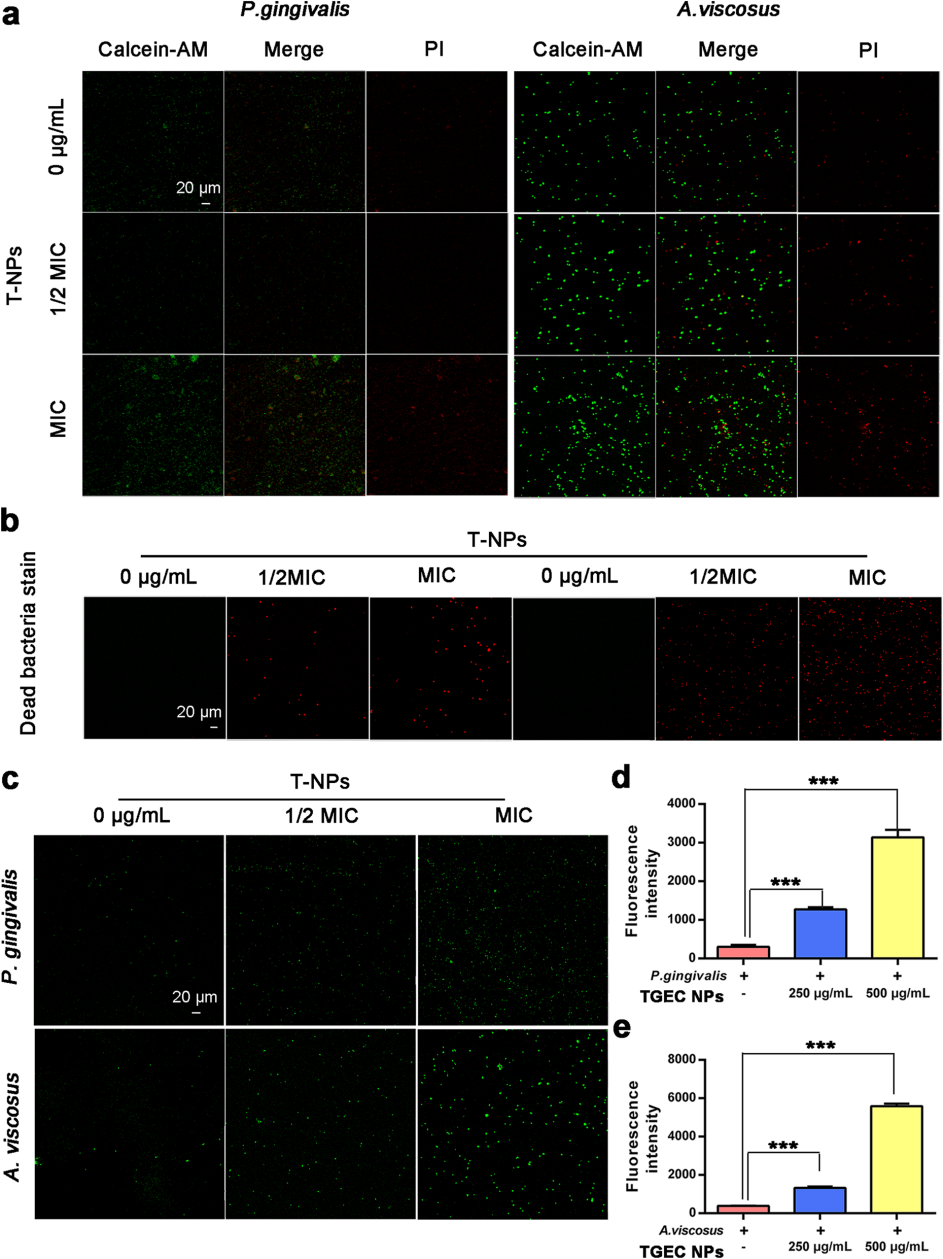
Live/dead differentiation of T-NPs on bacteria and intracellular ROS generation analysis. **a** Fluorescence images of live and dead *P. gingivalis* and *A. viscosus* cultured in the TSB medium with 1/2 MIC T-NPs, MIC T-NPs or without T-NPs and stained with calcein-AM and PI. **b** a group of fluorescence images of dead bacteria. Fluorescence images were captured under the excitation of 488 and 552 nm, respectively. And red dots represented dead bacteria and green dots represent live bacteria. **c** Fluorescence images of *P. gingivalis* and *A. viscosus* cultured in the TSB medium with 1/2 MIC T-NPs, MIC T-NPs or without T-NPs and incubated with DCFH-DA. Fluorescence images were captured under the excitation of 488 nm. **d** The fluorescence intensity of ROS in *P. gingivalis*. **e** The fluorescence intensity of ROS in *A. viscosus* was quantified

PI and calcein-AM were used to conduct confocal imaging studies on bacteria treated with different concentrations of T-NPs to reveal the interaction of T-NPs with *P. gingivalis* and *A. viscosus*. Most live *P. gingivalis* and *A. viscosus* were observed in green fluorescence and red fluorescence with no drug treat. In contrast, the observed red fluorescence was significantly increased after treated with T-NPs (Fig. 7a), indicating that most of bacterial cells were disrupted or lysed without any integrated morphology, and thus inhibit bacterial growth. In order to be more intuitive to observe the increase of the dead bacteria, a group of red fluorescence images of dead bacteria was shown in Fig. 7b. ROS such as superoxide ions (O_2_^−^), hydrogen peroxide (H_2_O_2_), and hydroxyl ions (OH^−^) produced by the interaction of TG-NPs, which could induce oxidative stress in bacteria. 2,7-dichlorofluorescein-diacetic acid (DCFH-DA) was used to study the generation of reactive oxygen species in bacteria in this study. Bacteria in control group did not emit green fluorescence, and laser illuminated treated cells produced bright green color depend on the presence of T-NPs, which confirmed propagation of ROS inside cells (Fig. 7c). The ROS fluorescence densities were quantified by Image J software and exhibited in Fig. 7d-e, bacterial cells treated with 2.5 mg/ml T-NPs, ROS fluorescence densities in *P. gingivalis* and *A. viscosus* were 4.32 and 3.40 times higher than ROS in the untreated group, respectively. Treated with 2.5 mg/ml T-NPs, ROS fluorescence densities in *P. gingivalis* and *A. viscosus* were 10.47 and 14.79 times higher than ROS in the untreated group, respectively. These findings indicated that T-NPs cause oxidative stress, which can damage bacterial cell membranes. Besides, intracellular ROS expression was dose dependent on T-NPs concentration. These results demonstrated that T-NPs interacted with the surface of bacterial cells to inhibit bacterial growth by inducing the production of ROS and leading to the destruction of bacterial mitochondrial structure. To conclude, the antibacterial activity was connected to the concentration of T-NPs, and the cell inhibitory mechanism might due to membrane disruption or ROS production.

## Discussion

Interestingly, a phenomenon in this work is that the intracellular ROS fluorescence intensity increases with the increasing concentrations of T-NPs. However, benefit from the combined polyphenols, T-NPs exhibited superior antioxidant activities in vitro*.* Is this contrary to its role in promoting ROS production in bacteria? Previous findings have demonstrated that excessive ROS could induce excessive host immunity, which might aggravate the level of inflammation. James E. Casanova et al. found that ROS was one of mechanisms that macrophages to kill bacteria and brain-specific angiogenesis inhibitor-I mediated bactericidal activity was dose-dependent with ROS to some extent. Therefore, they had linked bacterial detection to the induction of oxidative death [34]. Recent studies indicated that nanomaterials could penetrate into cells, promote the production of ROS, and damage bacterial cell membranes [35]. Therefore, we hypothesized that unlike in vitro experiment, polyphenols bind to protein sites after T-NPs enter the bacteria and promote the excessive production of ROS, thus inducing bacteria lysis and death. These results revealed an interesting phenomenon that T-NPs may play a potential therapeutic role from alleviating the periodontal tissue damage caused by excessive oxidation reaction and inhibiting the growth of pathogenic bacteria.

The construction of functional supramolecular polymerization systems using small or high molecular compounds has always been the focus of attention [36]. A phenomenon of small-sized NPs self-assembling into a pomegranate type particle had attracted our attention. Here, we proposed a hypothesis that the size increased of T-NPs during experiment process may be due to TG effective constituent oxidative self-polymerized to form smaller size particles. These dispersed particles cannot be connected to each other to form regular morphology. By controlling the reaction time, slightly larger particles are formed, which show strong aggregation and spontaneously form larger micro-spheres. Moreover, the release rate of T-NPs was rapid in the first 24 h at pH 8.0, which might rely on the special pomegranate structure of the particles. The bio-mimetic and functional properties of polyphenol supramolecular self-assembly made it has a broad application prospect in the field of medicine.

## Conclusions

A simple method for the preparation of microspheres from natural polyphenols was developed, which provided a useful reference for one-step prepared drug carriers from effective natural product components and offered a green and effective solution for treatment of gingivitis associated with periodontal pockets. In summary, a pH-sensitive and thermos-sensitive antibiotic platform based on natural polyphenols was constructed by the incorporation of T-NPs and poloxamers for effectively inhibiting bacterial associated with periodontitis. Owing to the pH sensitive of T-NPs and thermos-sensitive of poloxamer hydrogel, the slow and controllable release of T-NPs in periodontitis lesions in situ could be achieved by encapsulating T-NPs into hydrogel. The injectable hydrogels were in situ formed, provided the basis for T-NPs to alleviate the oxidative stress-induced periodontal tissue damage, inhibit the growth of oral pathogen, reduce the adhesion of bacteria, and enhance the retention of released drug in local infectious sites. This antibiotic platform exerted excellent prospects in treating local bacterial infection and protection of inflammatory diseases, such as adjuvant therapy for periodontitis. And it is expected to expand the use of natural phenolic compounds in other biomedical fields.

## Material and methods

### Materials

Ethanol, ammonia, K_2_S_2_O_8_, K_3_Fe(CN)_6_, trichloroacetic acid (TCA) and FeCl_3_ were analytical grade and purchased from Yongshen Chemical Reagent Co., Ltd. (Tianjin, China). Turkish Galls (*Quercus infectoria* Oliv.) was purchased from Anguo Medicinal materials Market, Hebei, China in October 2018. The insect gall was identified by Professor Bo Han (School of Pharmacy, Shihezi University).

### T-NPs preparation

#### TGEC eluted with 15% ethanol fraction from extract

TG were pulverized into fine powder and passed through a 60-mesh nylon sieve. 100.00 g of the powder was accurately weighed and extracted 3 times with 300 mL distilled water under reflux for 2 h. The combined filtrates were concentrated under the reduced pressure to yield the extract 78.0 g.

#### LC-MS analysis of TGEC

The samples were diluted to 1.0 mg/mL with distilled water and filtered through a 0.22 μm water film for HPLC-MS system analysis. X Bridge C18 column (*Ф*4.8 mm × 250 mm, 5 μm) (Waters, Milford, MA) was used for analysis of TGEC. The sample was eluted in a gradient elution program of A (0.1% Formic acid solution) and solvent B (acetonitrile): 7–7% B at 0–8 min, 7–20% B at 8–35 min, 20–30% B at 35–50 min, 30–50% B at 50–60 min. The injection volume was 20.0 μL and the sample was eluted at a flow rate of 1 mL/min. The detection wavelength was set at 269 nm. Mass system was equipped with an ACQUITY QDa source. Samples were analyzed in both positive and negative modes. ESI-MS conditions: desolvation temperature: 250 °C; source temperature: 120 °C; capillary voltage: 2.8 kV; extractor voltage: 50 V. Full scan spectra was obtained in the range of *m/z* 100 ~ 1250.

#### Synthesis of T-NPs

T-NPs were prepared by a classical Stöber method [37]. Typically, a mixture of ethanol (42.00 mL), water (18.00 mL) and NH_4_OH (13 mol/L, 1.80 mL) was stirred at room temperature for 30 min. TGEC (0.500 g) was precision weighing and brought to volume in a 10 mL flask with water, added into the mixture, and the reaction was stirred for 24 h. Sample was isolated via centrifugation at 3780×*g* for 10 min, washed for 3 times with ethanol, and dried overnight.

To elucidate the synthesis mechanism of T-NPs, Gallic acid was used as model drug for preparing Gallic acid NPs (GA-NPs) by the same method and preliminary analyzed the polymerization mechanism.

#### Synthesis of thermosensitive hydrogel loaded with T-NPs

A poloxamer gel was prepared by cold method. Briefly, different proportions of F127 (21 ~ 25%, w/v) and F68 (1%, w/v) were dissolved in deionized water and stored at 4 °C over night until completely dissolved, a clear hydrogel solution was obtained. The gelation time of thermosensitive hydrogel was determined by test tube inversion [38]. 2.00 mL of hydrogels were added to a tube (10.00 mL) and incubated in a water bath at 37.0 ± 0.5 °C. The gelation point is the time required for the hydrogel to become stagnant in the inverted tube. Based on the gelation was achieved in 1 min after mixing at 37.0 ± 0.5 °C, the suitable gel formula was selected for subsequent experiments. 20.00 mg of T-NPs were slowly added to 1.00 mL of hydrogel solution with continuous stirring until T-NPs were uniformly dispersed in the hydrogel solution.

### Characterization of T-NPs and hydrogel loaded with T-NPs

The morphology of T-NPs dispersed in water was observed by FE-SEM and analyzed by Image J software. The size distribution and Zeta potential were measured by Malvern laser granulator. By scanning in the range of 200 ~ 800 nm, the changes of peak wavelength of TGEC and T-NPs were investigated by UV-Vis spectrophotometry. TGEC, T-NPs and hydrogel and hydrogel loaded with T-NPs were dried and processed using a KBr pelletizing method. The infrared spectrums of samples were measured in the range of 4000 ~ 500 cm^− 1^. By analyzing peak position and peak type of infrared spectrum, the structure information on T-NPs was obtained. The morphology of hydrogel and hydrogel loaded with T-NPs were observed by SEM at 10 kV.

### In vitro studies: release of T-NPs and hydrogel loaded with T-NPs

Gradient concentrations of T-NPs were prepared and determined using a Microplate Reader at 350 nm. And the concentration-OD standard curve was calculated and drew. 1.00 mL T-NPs contains 9.95 mg solid content were added into dialysis bag and dialyzed against 200.00 mL artificial saliva at pH 5.5, 6.8 and 8.0 under magnetic stirring (100 r/min) in the beaker. The concentration of T-NPs suspension was indirectly determined at 350 nm, and deionized water was placed in a dialysis bag as control. The temperature was maintained at 37.0 ± 0.5 °C. At 0, 2, 4, 8, 12, 16, 20, 24 h, 1.00 mL samples were withdrawn and replaced with 1.00 mL fresh artificial saliva with the same pH value according to the principle of total volume invariably. Similar to the method used to determine the release of T-NPs, 1.00 mL of hydrogel loaded with 20.00 mg T-NPs solution was added into dialysis bag, but there is a slight difference, samples were withdrawn at 0, 2, 4, 8, 12, 16, 20, 24, 36, 48, 60, 72, 84, 96 h, 1.00 mL.

The absorbance value of samples was determined. The cumulative release rate was calculated using the formula 1. The release curves were plotted as a percentage of samples released versus time.

1 $$\mathrm{Cumulative}\ \mathrm{release}\ \mathrm{rate}\ \left(\%\right)=\left[\frac{C_n}{L/{V}_2}+\frac{\frac{\left({C}_{n-1}+\cdot \cdots \cdots +{C}_2+{C}_1\right)}{L/{V}_2}\times {V}_1}{V_2}\right]\times 100\%$$

*C*_*n*_ represented T-NPs concentrations in the sample taken at the designed time points; *L* represented theoretical drug dosage; *V*_*1*_ represented the sample volume taken at the designed time points; and *V*_*2*_ represented the total volume of the release medium.

### Performances of hydrogel loaded with T-NPs

#### Swelling behavior

The pre-weighed dry hydrogel sample was completely immersed artificial saliva at pH values of 4.5, 6.8 and 8.0 respectively and kept immersed at room temperature.

Over certain time interval, the swollen hydrogel sample was taken out of the solution, and the excess water was removed with a filter, and accurately weighed. The measurements were continued until the swollen hydrogel sample reached a constant weight. Equilibrium swelling (ES) was calculated using the formula 2:

2 $$\mathrm{ES}\ \%=\frac{\left({W}_t-{W}_d\right)}{W_d}\times 100\%$$

*W*_*t*_represented the weight of swollen hydrogel sample that removes surface moisture; *W*_*d*_represented the weight of dry hydrogel sample.

#### Viscosity measurements and rheological analysis

The viscosity of the T-NPs loaded hydrogel was measured using a digital viscometer at 25 °C to 40 °C. The instrument was kept in a horizontal level after installation and the appropriate rotor was selected according to the viscosity to be measured, getting the reading directly.

The rheological properties of T-NPs loaded hydrogel were investigated though an Anton Paar rheometer at 37.0 ± 0.5 °C, where the diameter of the two parallel plates is 25 mm and the gap between two parallel plates is 1 mm. The hydrogel samples subjected to the frequency sweep were measured for storage modulus (*G*′) and loss modulus (*G″*) at a constant strain (*γ* = 0.1%), to study viscoelastic behavior of the hydrogel samples at a wide range of angular frequencies (0.1 ~ 100 Hz).

### Anti-oxidation activity determination of T-NPs

#### DPPH radical scavenging assay

The DPPH radical scavenging activity of T-NPs was slightly modified according to the reported methods [39]. In brief, 50.0 μL sample solution with a proportional range (0.01, 0.02, 0.04, 0.08, 0.16, 0.32, and 0.64 mg/mL) was mixed with 25.0 μL DPPH solution (0.4 mM) and 100.0 μL deionized water. The absorbance at 517 nm was measured by UV-Vis spectra after the solutions were incubated in the dark at room temperature for 30 min. Ascorbic acid (VC) was used as positive control. The capacity of DPPH radical scavenging activity was calculated using the formula 3:

3 $$\mathrm{DPPH}\ \mathrm{radical}\ \mathrm{scavenging}\ \mathrm{activity}\ \left(\%\right)=\left(1-\frac{Abs_1-{Abs}_2}{Abs_0}\right)\times 100\%$$

*Abs*_*0*_ represented Abs of the blank control (replacing sample with water). *Abs*_*1*_ represented Abs of T-NPs or VC, and *Abs*_*2*_ represented Abs of the sample, which was the same as *Abs*_*1*_ and replaced the DPPH solution with methanol.

#### ABTS radical scavenging assay

The ABTS radical scavenging activity of T-NPs was evaluated. In short, ABTS radical solution was pregenerated by mixing 4.95 mM potassium persulfate and 7.0 mM ABTS in the dark at room temperature for 12 h until the reaction was complete and the absorbance was stable. The absorbance of ABTS radical solution was adjusted to 0.70 ± 0.02 by diluting with distilled water at 734 nm. 200 μL ABTS solution was mixed different concentration of the sample solution (0.01, 0.02, 0.04, 0.08, 0.16, 0.32, and 0.64 mg/mL), and the Abs was measured at 734 nm after the solutions were incubated in the dark at room temperature for 6 min. VC was used as a positive control. The capacity of ABTS radical scavenging activity was calculated using the formula 4:

4 $$\mathrm{ABTS}\ \mathrm{radical}\ \mathrm{scavenging}\ \mathrm{activity}\ \left(\%\right)=\left(1-\frac{Abs_1-{Abs}_2}{Abs_0}\right)\times 100\%$$

*Abs*_*0*_ represented Abs of the blank control (replacing sample with water), *Abs*_*1*_ represented Abs of T-NPs or VC, and *Abs*_*2*_ represented Abs of the sample only (replacing ABTS with PBS).

#### Reducing power assay

In the reducing power determination, 1.00 mL sample solutions with a proportional range (0.01, 0.02, 0.04, 0.08, 0.16, 0.32, and 0.64 mg/mL) were mixed with 2.50 mL of phosphate buffer (0.20 M, pH 6.6) and 2.50 mL [K_3_Fe(CN)_6_] (*w/v*, 1%). Mixture was placed in a water bath at 50 °C for 20 min. 2.50 mL trichloroacetic acid solution (10%, *w/v*) was added to the mixture and centrifuged at 530×*g* for 10 min. 100.0 μL supernatant was mixed with 100.0 μL distilled water and 20.0 μL FeCl_3_ solution (0.1%, *w/v*) for 10 min. The absorbance was measured at 700 nm.

### Antibacterial activity assay of T-NPs

#### Minimum inhibitory concentration (MIC) and IC_50_ determination

MIC assay was carried out using the two-fold microdilution broth method. Briefly, *P. gingivalis* or *A. viscosus* were pre-cultured in Trypticase Soy Broth (TSB) medium for 16 ~ 20 h. The cells (10^6^ CFU/mL, 100 μL) were mixed with serial dilutions of T-NPs suspension (10.00 mg/mL, 100 μL) in 96-well microplate by double dilution. After incubation at 37.0 °C for 24 h in anaerobic conditions containing 80% N_2_, 10% CO_2_, 10% H_2_, cell growth were detected by microplate reader. MIC was defined as the lowest concentration of the sample inhibiting visible growth of bacteria, and IC_50_ was calculated as the half-maximal inhibitory concentrations.

#### Inhibition zone determination

The Oxford cup method was used to determine the antimicrobial activity of T-NPs on the growth of *P. gingivalis* or *A. viscosus* The bacterial (10^6^ CFU/mL, 150 μL) was “flood inoculated” onto the surface of the medium. The oxford cups (6 mm in diameter) for inoculation of the samples were placed on the microbial inoculated agar plates. T-NPs was suspended in fresh TSB medium to a final concentration of 5.00 mg/mL and filtered through 0.22 μm hydrophobic membranes. 200.0 μL the sample was added to each oxford cup. In addition, metronidazole (0.50 mg/mL) was used as the positive control. The petri dishes were first diffused at 4 °C for 4 h and incubated at 37.0 °C for 24 h. Each experiment was repeated 3 times. And then the antagonistic activity was estimated by the diameters of the inhibition zone.

#### Morphology effects of T-NPs on bacteria

Morphological changes of *P. gingivalis* and *A. viscosus* were observed by FE-SEM. The bacteria grown in the presence, T-NPs (MIC and 1/2 MIC) were added to each well and cultivated for 4 h. Bacteria were obtained by centrifugal separation at 530×*g* for 3 min and washed several times with PBS. Suspension was fixed using 2.5% glutaraldehyde in PBS for 4 h at 4 °C. Bacteria were washed with PBS and post-fixed in 2% osmium tetroxide in PBS for 30 min and dehydrated in 30, 50, 70, 80, 90, 95 and 100% for 10 min each time. *P. gingivalis* and *A. viscosus* cultured with TSB medium were used as control, respectively. Samples were completely dried and coated with gold by sputtering for imaging and analysis by FE-SEM.

#### Live/dead bacteria stain

The bacteria grown in the presence and were added into 96-well plates, T-NPs (MIC and 1/2 MIC) were added to each well and cultivated for 4 h. Bacteria were collected and washed with phosphate buffered solution (PBS) for 3 times and re-suspended in PBS. To compare the microbial live/dead differentiation performance, bacteria suspension was stained with the mixture of propidium iodide (PI) solution (4.5 μM) and calcein-AM solution (2 μM) for 30 min in 37.0 °C. The stained bacteria were imaged using laser scanning confocal microscope (LSCM). The excitation maxima for Calcein-AM/PI are 490 nm.

#### Intracellular reactive oxygen species (ROS) detection

ROS including superoxide ions (O_2_^−^), hydrogen peroxide (H_2_O_2_) and hydroxyl ions (OH^−^) were produced by the interaction of bacteria with T-NPs. T-NPs induced the production of ROS from bacterial. To visualize the effect of T-NPs, bacteria were inoculating in a 24-well plate and cultivated for 20 h, T-NPs (MIC or 1/2 MIC) was added to wells and cultivated for 4 h. The cells were mixed with 10 μM DCFH-DA (2,7-Dichlorodi-hydrofluorescein diacetate) and incubated at 37.0 °C for 20 min to visualize the ROS production in treated cells. Samples were washed with PBS for 3 times, the fluorescence images were performed using LSCM at an excitation wavelength of 488 nm. Fluorescence signal intensity was quantified via Image J software.

### Statistical analysis

Statistical analysis was performed using SPSS version 22.0. One-way analysis of variance (ANOVA) was used to determine vital differences between groups, the data were expressed as mean ± *SD* and the threshold for significant differences was set at *p* < 0.05, *p* < 0.01, *p* < 0.001, respectively.

## Supplementary Information

**Additional file 1: Supplementary Figure S1.** LC-MS chromatogram of GA NPs collected in different point-in-time of reaction. **Supplementary Figure S2.** Potential of T-NPs under different pH conditions of artificial saliva. **Supplementary Video S1.** The forming process of TGEC NPs.

## Data Availability

All data needed to evaluate the conclusions in the paper are present in the paper and/or the Supplementary Materials.

## Abbreviations

TG: Turkish gall
TGEC or T: Turkish Galls effective constituent
T-NPs: Turkish Galls effective constituent nanoparticles
GA-NPs: Gallic acid nanoparticles
MIC: Minimum inhibitory concentration
ROS: Reactive oxygen species
NSAID: Nonsteroidal Antiinflammatory Drug
*S. aureus*: *Staphylococcus aureus*
F127: Poloxamer 407
F68: Poloxamer 188
VC: Vinylene carbonate
*A. viscosus*: *Actinomyces viscosus*
*P. gingivalis*: *Porphyromonas gingivalis*
PI: Propidium Iodide
Calcein-AM: Calcein acetoxymethyl ester
DCFH-DA: 2,7-dichlorofluorescein-diacetic acid
DPPH: 1,1-Diphenyl-2-picrylhydrazyl radical 2,2-Diphenyl-1-(2,4,6-trinitrophenyl)hydrazyl
ABTS: 2,2ˊ-Azino-bis-(3-ethylbenzothiazoline-6-sulfonic acid) diammonium salt
