# Supplementary material for: Natural polyphenol self-assembled pH-responsive nanoparticles loaded into reversible hydrogel to inhibit oral bacterial activity
Source: Mol Biomed. 2022 Sep 16;3:28. doi: 10.1186/s43556-022-00082-3 (PMC9478017; doi:10.1186/s43556-022-00082-3)
Supplement: Supplementary file 1 — Additional file 1: Supplementary Figure S1. LC-MS chromatogram of GA NPs collected in different point-in-time of reaction. Supplementary Figure S2. Potential of T-NPs under different pH conditions of artificial saliva. Supplementary Video S1. The forming process of TGEC NPs. [file 43556_2022_82_MOESM1_ESM.zip › Support information .docx]

**Supporting Information**

**Smart Hydrogel Loaded with** **Natural Polyphenol Self-assembled Nanoparticles Effectively Inhibits Bacterial Associated with Periodontitis**

Yunyun Qi ^†^, Jinxiang Yang ^†^, Yaping Chi, Peng Wen, Shiyi Yu, Luhui Liu, Zhongying Wang, Rui Xue, Yan Zhang and Bo Han^^[[1]](#footnote-0)^^

School of Pharmacy/Key Laboratory of Xinjiang Phytomedicine Resource and Utilization, Ministry of Education, Shihezi University, Shihezi 832002, P. R. China.

^[[2]](#footnote-1)^

**Supplemental figure legend**

**Figure S1** LC-MS chromatogram of GA NPs collected in different point-in-time of reaction. **a** LC-MS chromatogram of GA. **b** LC-MS chromatogram of GA NPs collected in 2 h of reaction. **c** LC-MS chromatogram of GA NPs collected in 4 h of reaction

**Figure S2.** Potential of T-NPs under different pH conditions of artificial saliva

**
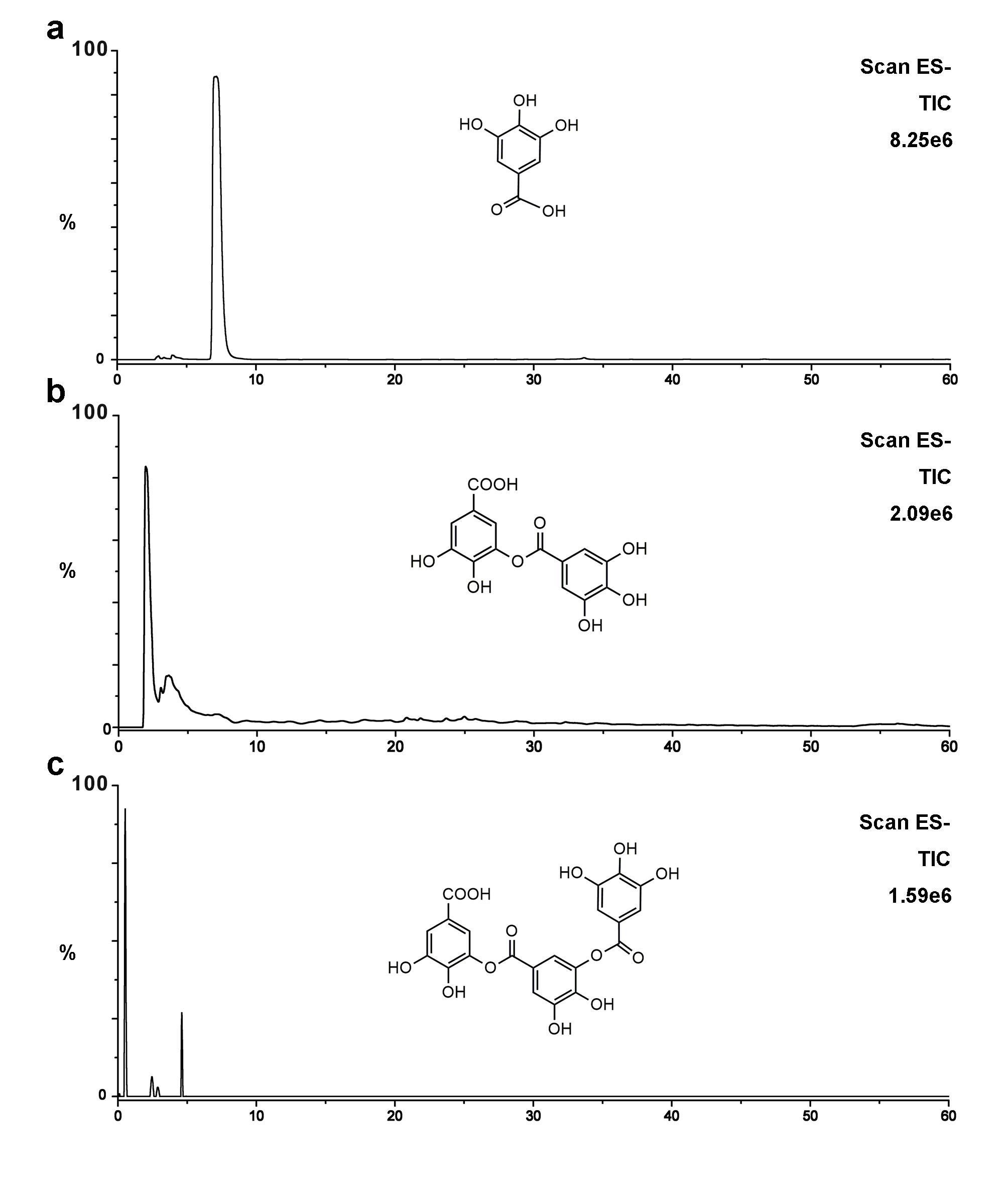
**

**Fig. S1**

**
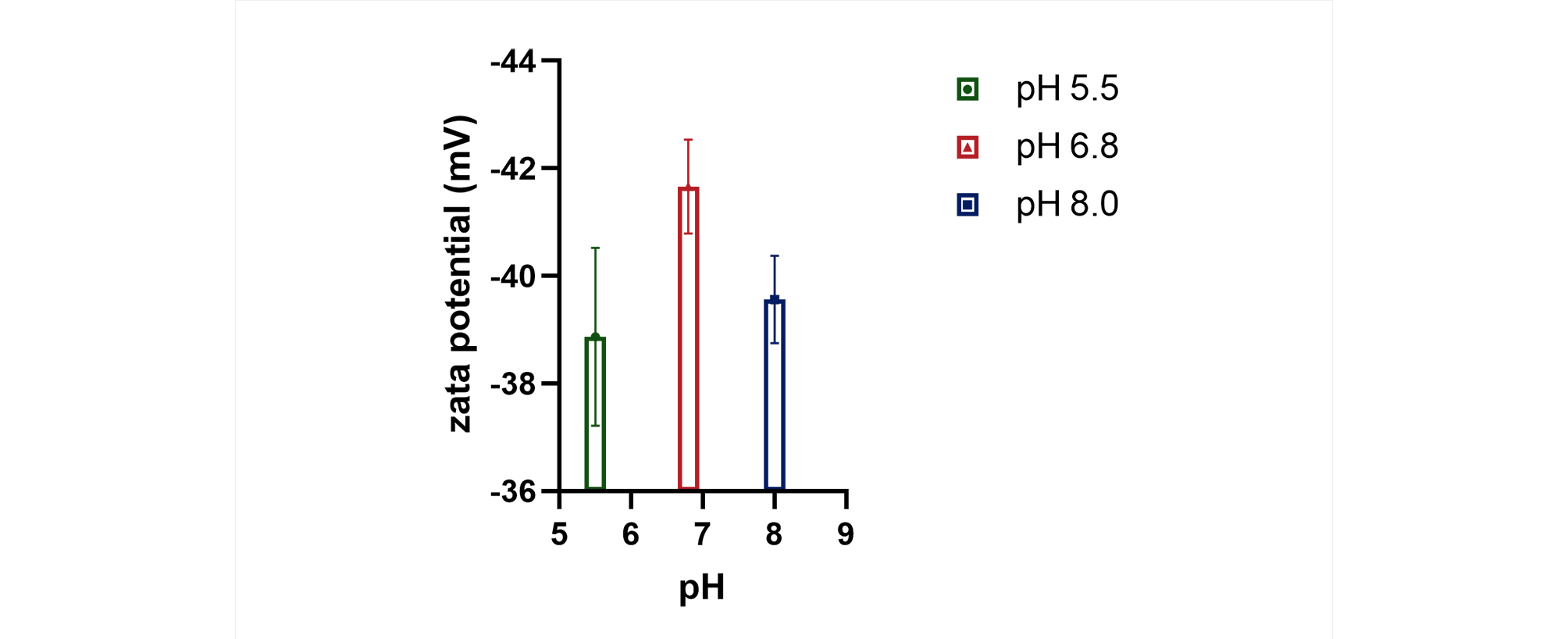
**

**Fig. S2**

**Supplemental video**

**Video S1** The forming process of TGEC NPs.

视频目前只能右键选择幻灯片对象播放。

**Supplemental materials**

***Microorganism*** *Porphyromonas gingivalis* (*P. gingivalis*, ATCC 33277) and *Actinomyces viscosus* (*A. viscosus*, ATCC 27044) were purchased from Nanjing Clinical Biological Technology Co., Ltd.

***Chemicals*** Formic acid (Lot#: C10101377) was purchased from Shengao Chemical Reagent Co., Ltd. (Tianjin, China). Methyl alcohol (Lot 151345, HPLC grade) and acetonitrile (Lot 178436, HPLC grade) were obtained from Fisher Scientific (Fair Lawn, NJ, USA). 1, 1-diphenyl-2-picrylhydrazyl (DPPH, Lot D9132) was purchased from Sigma-Aldrich Co., Ltd. (St. Louis, Missouri, USA). 2, 2'-azino-bis (3-ethylbenzothiazoline-6-sulfonic acid) (ABTS, Lot 180305) was purchased from Lanji biology Co., Ltd (Shanghai, China). Poloxamer 407 (F127, WPAK588B) and poloxamer 188 (F68, WPAK527B) were purchased from Fengli Jingqiu Pharmaceutical Co., Ltd. (Beijing, China).

### *Instruments.* The instruments used here includes Waters X EVO TQD HPLC-MS system (LC-MS, Massachusetts, U.S.A.), FA2014B electronic balance (Shanghai precision scientific instruments Co., Ltd, China), RPB-10 pen type pH meter, HJ-3 constant temperature magnetic stirrer (Changzhou Guohua Electric Appliance Co., Ltd), TDL-5M Table Model High Speed Centrifuge (Sichuan Shuke Instrument Co., Ltd, China), UV­­­‑2600 UV‑Vis spectrophotometer (Shimadzu Enterprise Management Co., Ltd, Japan), Milli-Q Integral water system (0.22 μm, Millipore, Bedford, U.S.A.), Fourier transform infrared spectrometer (FT-IR, Shimadzu Enterprise Management Co., Ltd, China), field emission-scanning electron microscope (FE-SEM, ZEISS SUPRA 55 VP, ZEISS LEO, Germany), BioTek Cytation 3 Cell Imaging Multi-Mode Reader (BioTek, Winooski, VT, USA), Anton Paar rheometer (MCR301, Germany), DV-C digital viscometer (Brookfield Engineering Laboratories, Inc., USA), Zeiss LSM 510 META Laser Scanning Confocal Microscope (LSCM, Zeiss, Germany) and Zeta sizer Nano S90 Marvin high-sensitive nanoparticle analyzer (British Malvern Instruments Limited).

1. * Corresponding Author:

   Bo Han (1982-), professor

   School of Pharmacy, Shihezi University, Xinjiang Shihezi, P. R. China

   Tel: +86 0993-2057005

   Fax: +86 0993-2057005

   E-mail address: hanbodexinxiang@163.com

   †These two authors equally contributed to this work. [↑](#footnote-ref-0)
2. [↑](#footnote-ref-1)
