# Supplementary figures and images for: Natural polyphenol self-assembled pH-responsive nanoparticles loaded into reversible hydrogel to inhibit oral bacterial activity
Source: Mol Biomed. 2022 Sep 16;3:28. doi: 10.1186/s43556-022-00082-3 (PMC9478017; doi:10.1186/s43556-022-00082-3)

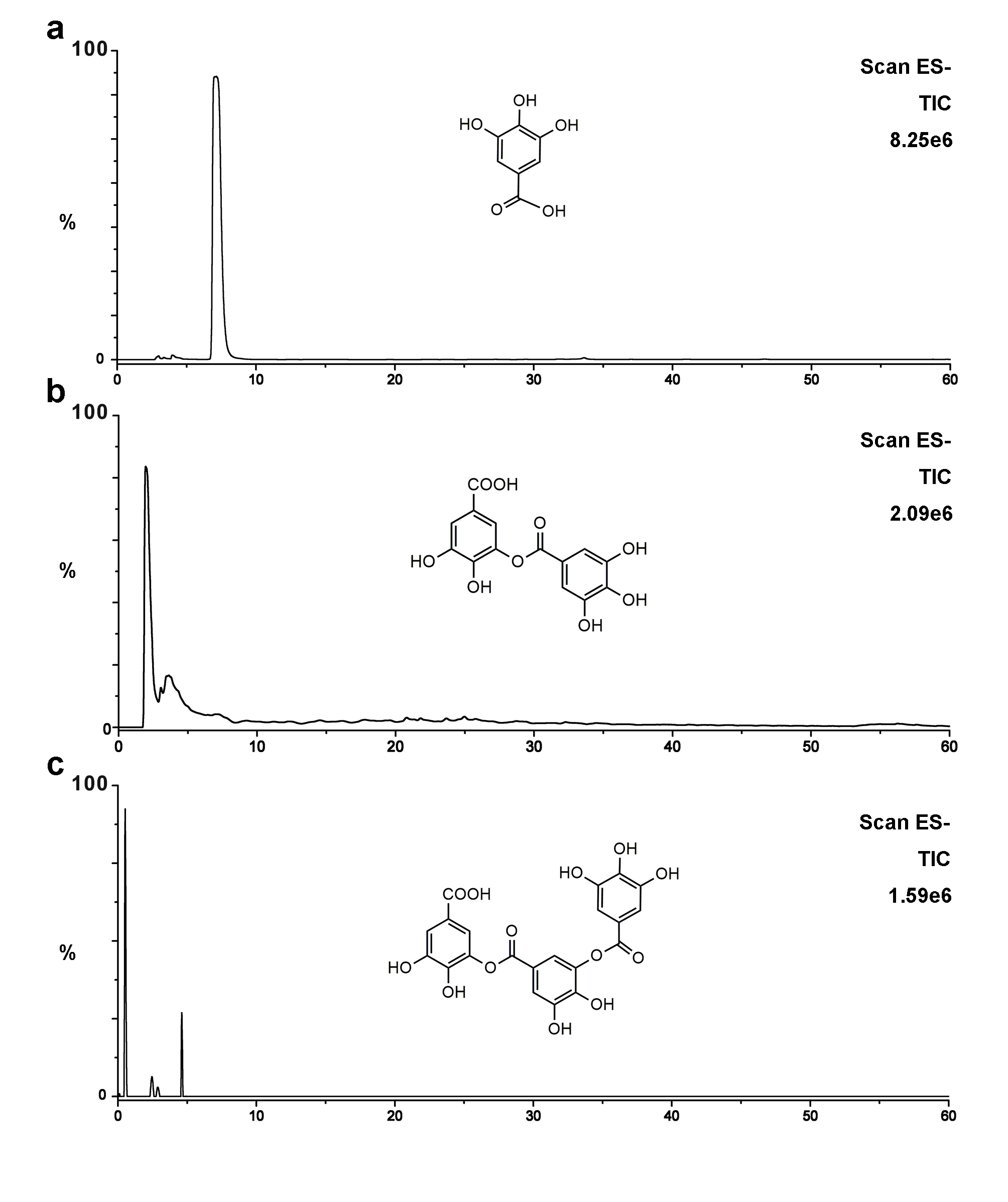

Supplement: Supplementary file 1 — Additional file 1: Supplementary Figure S1. LC-MS chromatogram of GA NPs collected in different point-in-time of reaction. Supplementary Figure S2. Potential of T-NPs under different pH conditions of artificial saliva. Supplementary Video S1. The forming process of TGEC NPs. [file 43556_2022_82_MOESM1_ESM.zip › fig.S1.tif]

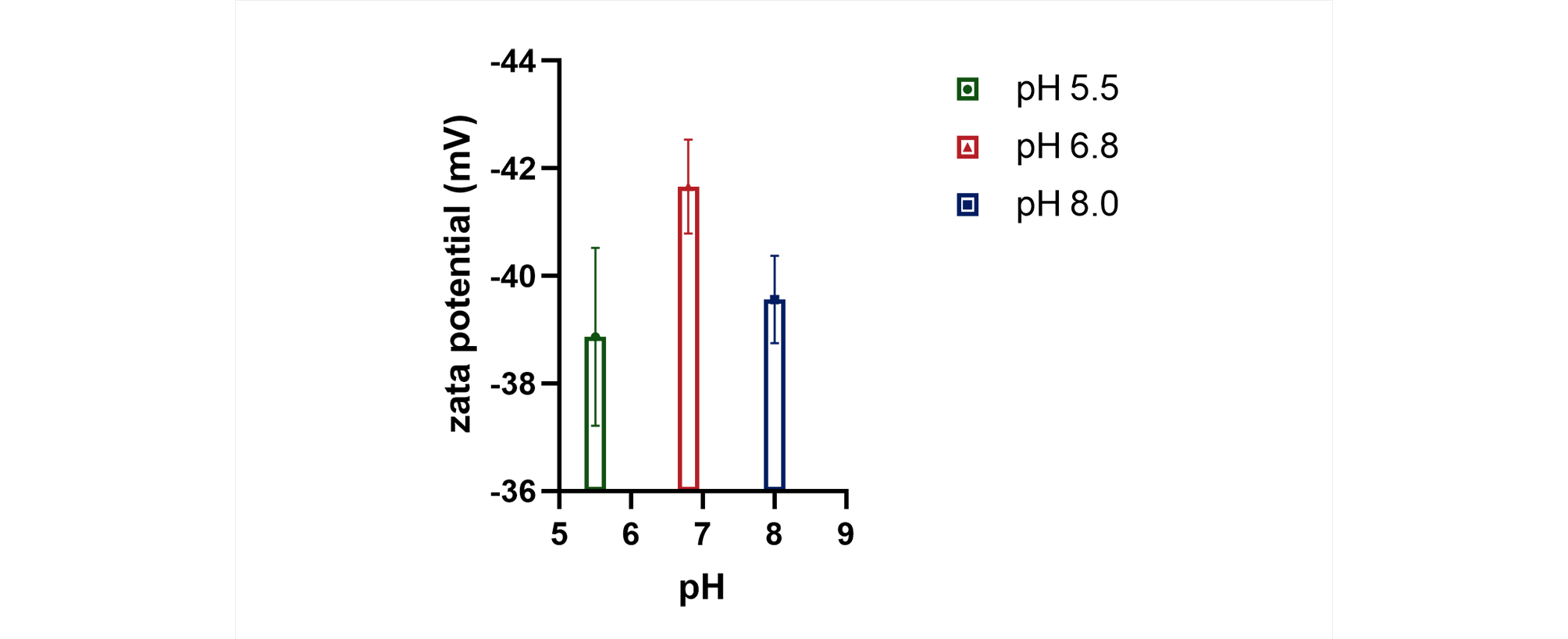

Supplement: Supplementary file 1 — Additional file 1: Supplementary Figure S1. LC-MS chromatogram of GA NPs collected in different point-in-time of reaction. Supplementary Figure S2. Potential of T-NPs under different pH conditions of artificial saliva. Supplementary Video S1. The forming process of TGEC NPs. [file 43556_2022_82_MOESM1_ESM.zip › fig.S2.tif]
